# Sequencing, genome analysis and prevalence of a cytorhabdovirus discovered in *Carica papaya*

**DOI:** 10.1371/journal.pone.0215798

**Published:** 2019-06-20

**Authors:** Andrés X. Medina-Salguero, Juan F. Cornejo-Franco, Samuel Grinstead, Dimitre Mollov, Joseph D. Mowery, Francisco Flores, Diego F. Quito-Avila

**Affiliations:** 1 Facultad de Ciencias de la Vida, Escuela Superior Politécnica del Litoral, ESPOL, Guayaquil, Guayas, Ecuador; 2 Centro de Investigaciones Biotecnológicas del Ecuador (CIBE), Escuela Superior Politécnica del Litoral, ESPOL, Guayaquil, Guayas, Ecuador; 3 National Germplasm Resources Laboratory, USDA-ARS, Beltsville, MD United States of America; 4 Electron and Confocal Microscopy Unit, USDA ARS, Beltsville, MD, United States of America; 5 Centro de Investigación de Alimentos, CIAL, Facultad de Ciencias de la Ingeniería e Industrias, Universidad Tecnológica Equinoccial-UTE, Quito, Pichincha, Ecuador; 6 Departamento de Ciencias de la Vida y la Agricultura, Universidad de las Fuerzas Armadas-ESPE, Sangolquí, Pichincha, Ecuador; China Agricultural University, CHINA

## Abstract

The complete genome of a new rhabdovirus infecting papaya (*Carica papaya* L.) in Ecuador, named papaya virus E, was sequenced and characterized. The negative-sense single-stranded RNA genome consists of 13,469 nucleotides with six canonical open reading frames (ORFs) and two accessory short ORFs predicted between ORFs corresponding to P3 (movement protein) and M (matrix protein). Phylogenetic analyses using amino acid sequences from the nucleocapsid, glycoprotein and polymerase, grouped the virus with members of the genus *Cytorhabdovirus*, with rice stripe mosaic virus, yerba mate chlorosis-associated virus and Colocasia bobone disease-associated virus as closest relatives. The 3’ leader and 5’ trailer sequences were 144 and 167 nt long, respectively, containing partially complementary motifs. The motif 3’-AUUCUUUUUG-5’, conserved across rhabdoviruses, was identified in all but one intergenic regions; whereas the motif 3’-ACAAAAACACA-5’ was found in three intergenic junctions. This is the first complete genome sequence of a cytorhabdovirus infecting papaya. The virus was prevalent in commercial plantings of Los Ríos, the most important papaya producing province of Ecuador. Recently, the genome sequence of bean-associated cytorhabdovirus was reported. The genome is 97% identical to that of papaya virus E, indicating that both should be considered strains of the same virus.

## Introduction

The *Rhabdoviridae*, a negative-sense RNA virus family, contains viruses that infect a wide range of hosts including vertebrates, invertebrates and plants [1]. Virions have a helical, bullet-shape morphology, surrounded by a host-derived membrane [2]. Rhabdovirus genomes range from 11 to 16 kilobases (kb) with only non-segmented ones classically assigned to genera in the family. However, virus species with bipartite genomes have recently been included in the *Rhabdoviridae* [3,4].

Based on host type, genomic organization and other biological features, rhabdoviruses currently are organized in 18 genera [5]. High-throughput sequencing (HTS) techniques have led to the discovery of several novel rhabdoviruses for which new genera have been proposed [6,7,8].

The genome organization of rhabdoviruses has a canonical arrangement of five genes: 3’-N-P-M-G-L-5’ that encode the nucleocapsid, phosphoprotein, matrix protein, glycoprotein and the large polymerase, respectively. Terminal regions have non-coding regulatory sequences denoted, respectively, as 3’-leader (*l*) and 5’ trailer (*t*) [9,10]. Additional “accessory” genes have been observed in arrangements that differ among rhabdoviruses [11].

Monopartite plant-infecting rhabdoviruses have long been classified into the genera *Cytorhabdovirus* or *Nucleorhabdovirus*, based on their cytoplasmic or nuclear, site of replication in the cell, respectively [9,12]. This biological feature has been confirmed by phylogenetic relationships, which separate clearly the two groups. Recently, two new genera, *Dichorhavirus* and *Varicosavirus*, have been created to classify bi-partite plant-infecting rhabdoviruses [3,4].

Although more than 80 monopartite plant rhabdoviruses have been reported, based on cytopathology studies, complete genomes are only available for a few members of each genus. A genomic feature common to both cyto- and nucleorhabdoviruses is the presence of an additional open reading frame (ORF) between the P and M genes. The product of this ORF is considered the movement protein (MP) as it has been demonstrated to have cell-to-cell movement function [13,14]. Additional ORFs have been identified in the genomes of some plant rhabdoviruses, resulting in variations of the canonical genomic organization [11,15].

Plant infecting rhabdoviruses have been found in a wide range of hosts including monocots such as rice, maize, wheat and barley, and dicots such as potato, lettuce, carrot and strawberry, among others [12].

In papaya (*Carica papaya* L.), the occurrence of putative nucleorhabdoviruses in commercial plantings in Venezuela, Florida and Mexico was documented as early as 1980 [16,17,18]. In Venezuela, the virus was observed in tissue collected from trees showing a range of symptoms including leaf yellowing, apical necrosis and plant death [16]. In Florida, the virus was associated with droopy necrosis, a disorder that included bending of the upper section of the crown with a bunchy appearance which, at later stages, developed into necrosis and plant death [17]. However, no genomic sequences for papaya rhabdoviruses have been reported.

This study reports the complete genome sequence of a new cytorhabdovirus, its genomic characterization and prevalence in papaya plantings of Ecuador.

## Materials and methods

### Virus source

In 2016, a papaya sentinel plant (cv. Sunrise), previously used as part of a study on the epidemiology of papaya virus Q (PpVQ) and its relationship with papaya ringspot virus (PRSV) [19,20], was maintained under greenhouse conditions for further investigation. The study was conducted in Los Ríos, the largest papaya producing province of Ecuador, where sentinel plants were scattered in a 2-year old field and monitored for four months. The selected plant was subjected to virus testing using the reverse-transcription (RT)-PCR assay described by Quito-Avila et al.[20]. The plant tested positive for PRSV but not for PpVQ. Additional viruses infecting this plant were further investigated using the approach described below.

### Sequencing and genome analyses

Total RNA was extracted from the selected plant using the protocol described by Quito-Avila, et al. [20]; followed by DNase treatment. Viral RNA was enriched by depleting plant rRNA. Preparation of Trueseq RNA library, followed by high-throughput sequencing (HTS) on a HiSeq 4000 Illumina platform (100 paired-end reads) was performed at Macrogen (South Korea). Sequence reads were trimmed and assembled into contigs by CLC Genomic Workbench 11 (Qiagen USA). Contigs were analyzed using BLASTx from the *National Center for Biotechnology Information* (NCBI).

HTS data was verified by Sanger sequencing of RT-PCR amplified overlapping fragments, which were generated by specific primers. Terminal sequences were confirmed by RACE using total RNA as a template for cDNA, and specific primers near the ends as recommended by the manufacturer (Life Technologies, USA). Sequence comparisons, alignments and prediction of open reading frames (ORFs) were done using Geneious R 11 (Biomatters, New Zealand), the NCBI conserved domain database [21] and the Swiss-model server [22].

### Transmission electron microscopy (TEM)

Leaf tissue was dissected into one mm pieces using a biopsy punch, fixed in 2% paraformaldehyde, 2.5% glutaraldehyde, 0.2% Tween-20, 0.5M Na cacodylate and processed in a Pelco BioWave microwave as previously described [23]. TEM grids were stained with 4% uranyl acetate for 10 min and 2% lead citrate for 5 min, and imaged at 80kV with a Hitachi HT-7700 transmission electron microscope (Hitachi High Tech America, Inc., Dallas, TX, USA).

### Phylogenetic analyses

Protein sequences corresponding to the nucleocapsid (N), glycoprotein (G) and polymerase (L) were downloaded from all the nucleo- and cytorhabdoviruses available in GenBank. In addition, cytorhabdovirus-like sequences annotated as part of the whitefly (*Bemisia tabaci*) genome (acc. numbers: KJ994255-KJ994264), were identified and included in the analysis. Amino acid sequences were aligned using structural information with Expresso [24]. The confidence of the multiple sequence alignments was measured with TCS [25], and unreliable alignment fragments were discarded. The best evolution model for each alignment was determined with MEGA7 [26] and used to build single gene and multi-locus phylogenies on BEAST v.1.8.4 [27]. Two chains of one million Markov Chain Monte Carlo (MCMC) were run for each protein and for the multi-locus concatenation. Convergence of the runs and effective sample size were observed in Tracer v.1.6. The two runs were combined with a 10% burn-in using LogCombiner and a consensus tree was built with TreeAnnotator [27].

### Virus detection and survey

A total of 180 papaya plants from commercial fields in five Ecuadorean provinces (Los Ríos, Guayas, Manabí, Santa Elena and Sucumbíos) were tested for the virus as described [20]. PCR was done using primers: (F) 5’- CGCAAAACTCGATTGTTCCG-3’ and (R) 5’-CCTGCTGATGATCCTATCTCC-3’, which amplify a 779 nt fragment spanning a portion of the 3’ leader and the nucleocapsid gene.

In addition, up to five positive samples from each location were used to amplify a fragment of the virus polymerase using primers: (F) 5’-GAGAAGTGGAACCTCAATTTCC-3’ and (R) 5’-CTGAAGAGAGAAGGGTCGGT-3’. Amplicons (860 nt long) were cloned and sequenced. Sequence comparisons were performed using ClustalW [28] to determine sequence variability based on geographic location.

The annealing temperature for both primer pairs used in PCR was set at 55C.

## Results

### Sequencing

A total of 32,375,528 paired-end 100 nt reads were obtained from the HTS cDNA library. These reads were assembled into 61,033 contigs. Twenty-three contigs were identified as associated with plant viruses. One contig >13 kb revealed similarities to cytorhabdoviruses. The remaining contigs showed homology to PRSV. In subsequent analysis using Geneious R11 (Biomatters, New Zealand) these contigs were assembled into a ~10 kb PRSV genome. Only about 75 thousand reads (0.23%) mapped to the cytorhabdovirus, while 3.8 million (11.68%) identified with the PRSV contig.

### Genome organization

**Fig 1:**
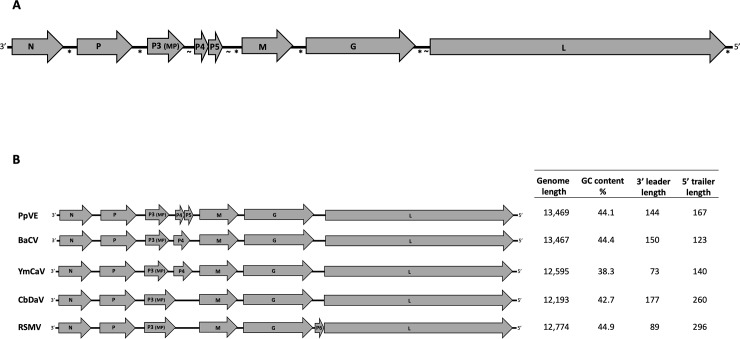
Genome organization of papaya virus E (PpVE) and closest relatives. (A) Open reading frames (ORFs), on the antigenomic strand (3’– 5’), of PpVE are illustrated by the grey arrows, where N represents the nucleocapsid, P the phosphoprotein, P3 (MP) the movement protein, M the matrix protein, G the glycoprotein and L the polymerase. Hypothetical proteins P4 and P5 are depicted as short contiguous ORFs. The presence of rhabdovirus conserved intergenic motif (3’-AUUCUUUUUG-5’) is denoted by the asterisk *. Junctions having PpVE conserved motif (3’-ACAAAAACACA-5’), are indicated by the symbol ~. (B) Genome organization comparison between PpVE and its closest relatives: bean-associated cytorhabdovirus (BaCV), yerba mate chlorotic associated virus (YmCaV), Colocasia bobone disease-associated virus (CBDaV) and rice stripe mosaic virus (RSMV). Genome features corresponding to each virus are provided on the right panel.

The entire genome of the new virus, provisionally named papaya virus E (PpVE) (GenBank accession no. MH282832) has 13,469 nt. The antigenomic strand contains six ORFs organized in the classical canonical order of monopartite plant rhabdoviruses, plus two short accessory ORFs (ORFs 4 and 5) (Fig 1).

ORFs 1, 2, 3 encode, respectively, the putative nucleocapsid (N), phosphoprotein (P) and movement protein (MP) P3. ORFs 4 and 5, arranged in a contiguous fashion with overlapping termination/initiation codons (e.g. UA**A****UG**), encode two small hypothetical proteins (P4 and P5, respectively) of unknown function. ORFs 6, 7 and 8 encode, respectively, the putative matrix protein (M), glycoprotein (G) and the polymerase (L) (Table 1).

**Table 1:** Predicted proteins of papaya virus E.

ORF			Predicted protein		
Number	Genome position (3’– 5’)	Length (nt)	Hypothetical function	Length (aa)	Predicted mass (kDa)
1	145–1,500	1,356	N	451	51.14
2	1,690–3,027	1,338	P	445	49.82
3	3,193–3,762	570	P3 (MP)	189	21.17
4	3,948–4,028	81	P4	26	2.69
5	4,028–4,114	87	P5	28	3.34
6	4,285–4,929	645	M	214	24.35
7	5,159–6,718	1,560	G	519	58.08
8	6,961–13,302	6,342	L	2113	241.29

The number and genome position of each open reading frame (ORF) are indicated. N: nucleocapsid, P: phosphoprotein, P3 (MP): movement protein, P4 (unknown hypothetical protein 4), P5 (unknown hypothetical protein 5), M: matrix, G: glycoprotein, L: polymerase.

PpVE has an overall nucleotide sequence identity of 97% with the genome of bean associated cytorhabdovirus (BaCV), which was found recently from common bean (*Phaseolus vulgaris* L.) in Brazil [29]. This identity level, according to the species demarcation criterium for cytorhabdoviruses [30], indicates that PpVE and BaCV are strains of the same virus.

BLASTx searches performed on each ORF of PpVE and BaCV revealed homology, with low sequence identities, to rhabdovirus proteins from different members of the genus *Cytorhabdovirus*, with rice stripe mosaic virus (RSMV), yerba mate chlorosis-associated virus (YmCaV) and Colocasia bobone disease-associated virus (CBDaV) as closest relatives [31,32,33] (Table 2). Genome organization comparison across closest relatives indicated that both BaCV and YmCaV contain an accessory ORF4; but lack the ORF 5, which is present in PpVE (Fig 1B). Conserved domain database and pfam searches [34] did not find orthologues for PpVE hypothetical proteins P 4 and 5.

**Table 2:** Amino acid sequence comparison between predicted proteins from papaya virus E and their counterparts from closest relatives.

	Identity (%)				
Protein	BaCV (MK202584)	RSMV (KX525586)	YmCaV (KY366322)	CBDaV (NC034551)	Virus-like sequences from whitefly ^a^ (KJ994255-60)
N	96.9	23	23	21	93
P	94.8	14	15	9	N.A.
P3 (MP)	96.8	15	23	16	99
P4	13.7	N.A.	7.7	N.A.	N.A.
P5	N.A.	N.A.	N.A.	N.A.	N.A.
M	98.6	9	11	15	N.A.
G	97.9	17	20	16	91
L	97.9	33	30	36	88–98

Percentage (%) identities are indicated for bean-associated cytorhabdovirus (BaCV), rice stripe mosaic virus (RSMV), yerba mate chlorosis associated virus (YmCaV), Colocasia bobone diasease-associated virus (CBDaV) and cytorhabdovirus-like sequences from whitefly genome. NCBI accession numbers, from which deduced proteins were obtained, are indicated below each virus acronym. N: nucleocapsid, P: phosphoprotein, P3(MP): movement protein, M: matrix, G: glycoprotein, L: polymerase, ORF: open reading frame, N.A. not applicable.

^a^ Partial rhabdovirus-like sequences annotated as part of the whitefly genome.

Amino acid sequence alignments between PpVE P4, and its counterpart from BaCV and YmCaV showed 7% and 13% identities, respectively (Table 2). However, when P4 and P5 of PpVE were concatenated for the alignment, amino acid sequence identitiy of 15% was observed between PpVE and the BaCV; and 20% between PpVE and YmCaV (S1 Fig), suggesting that PpVE proteins P4 and P5 might be translated as a single fused protein via a reinitiation translation mechanism (RTM), as reported for other rhabdoviruses [35,6].

### Intergenic regions

PpVE intergenic regions have an average of 36% GC content, except for the P3-P4 junction, which has an unusual 46.5%. The conserved motif 3’-AUUCUUUUUG-5’, was found at each ORF junction of PpVE, except for the P3-P4 junction, and also at the trailer region. This motif was fully conserved in gene junctions of PpVE closest relatives: BaCV, YmCaV, CBDaC and RSMV. Interestingly, a novel motif, with the core sequence 3’-ACAAAAACACA-5’, was identified in junctions P3-P4, P5-M and G-L of PpVE (Fig 1A). This motif was not found at junctions of PpVE closest relatives; but was partially conserved in one or two intergenic junctions of more distantly related cyto- and nucleorhabdoviruses (Table 3), suggesting a potential role in transcription regulation.

**Table 3:** Papaya virus E (PpVE) novel intergenic motif and its comparison with partially conserved counterparts from cyto- and nucleorhabdoviruses.

	Virus	Motif sequence	Location (Intergenic junction(s) or trailer)
**Cytorhabdoviruses**	PpVE	3-ACAAAAACACA-5	P3-P4, P5-M and G-L
	BaCV	3-ACAAAA*U*CACA-5	P4-M
		n3-A*C*AACAACACA-5	G-L
	MYSV	3-ACAAAA*U*CACA-5	P4-P6
	ADV	3-ACAAAA*CU*ACA-5	MP-M
	RSMV	3-A*G*AAA*C*ACACA-5	N-P
	CBDaV	3-ACAAA*G*ACA*G*A-5	P-MP
	LNYV	3-ACAAAA*U*C*U*CA-5	M-G
	SCV	3-AC*U*AAA*C*CACA-5	N-P
		3-ACAA*U*AAC*C*CA-5	MP-M
		3-ACA*GC*AACACA-5	M-G
		3-ACAAAAA*U*A*U*A-5	5’trailer
**Nucleorhabdoviruses**	EMDV	3-ACA*C*A*U*ACACA-5	P-MP
		3-A*U*AA*U*AACACA-5	M-G
	PhCMV	3-ACAA*U*AA*U*ACA-5	P-MP
	BCNRV-1	3-ACAA*C*AACAC*G*-5	MP-M
		3-ACA*C*AAA*G*ACA-5	5’trailer
	SYNV	3-ACA*CC*AACACA-5	MP-M
	MMV	3-ACAA*C*AACA*A*A-5	N-P

Bold-underlined nucleotides represent mismatches to the reference PpVE motif. N: nucleocapsid, P: phosphoprotein, MP: movement protein, M: matrix, G: glycoprotein, L: polymerase, ORF: open reading frame. Virus abbreviations: bean-associated cytorhabdovirus (BaCV), maize yellow striate virus (MYSV), alfalfa dwarf virus (ADV), rice stripe mosaic virus (RSMV), Colocasia bobone disease associated virus (CBDaV), lettuce necrotic yellows virus (LNYV), strawberry crinkle virus (SCV), eggplant mottled dwarf virus (EMDV), physostegia chlorotic mottle virus, blackcurrant nucleorhabdovirus 1 (BCNRV-1), sonchus yellow net virus (SYNV) and maize mosaic virus (MMV).

### Terminal regions

The 3’ leader of PpVE contains 144 nucleotides. This length is similar to those from distantly related cytorhabdoviruses such as maize yellow striate virus (MYSV, 143 nt), northern cereal mosaic virus (NCMV, 141 nt) and strawberry crinkle virus (SCV, 147 nt). Interestingly, the two most closely related cytorhabdoviruses, RSMV and YmCaV, have shorter 3’ leaders. The 5’ trailer of PpVE is 167 nt long, similar in size to its counterpart from YmCaV (Fig 1B).

Several motifs were identified in both the leader and trailer regions of PpVE, which are fully or partially complementary to each other. For instance, motifs 3’-GAUAAAA-5’ and 3’-CUAUUUU-5’ located at nt positions 12–18 and 13,430–13,436, respectively, complement each other, as reported for terminal regions of rhabdoviruses [6,10]. In addition, the motif 3’-**UUCUUUU**AA-5’ was identified at both the leader (nt 62–70) and trailer (nt 13,435–13,443) regions.

### Phylogenetic relationships

The multiple sequence alignments for N, G and L proteins were 831, 936, and 3,071 amino acids (aa) long, respectively. After eliminating ambiguous fragments, the resulting alignment lengths were 284, 269, and 1,692 aa for each protein, respectively. The best evolution model for N and L was LG+G [36]; while WAG+G+I [37] was the best model for the glycoprotein. Convergence of the runs and effective sample size above 200 was observed for all the parameters that were inferred in Bayesian analyses, indicating that the estimation of their posterior distribution is reliable.

**Fig 2:**
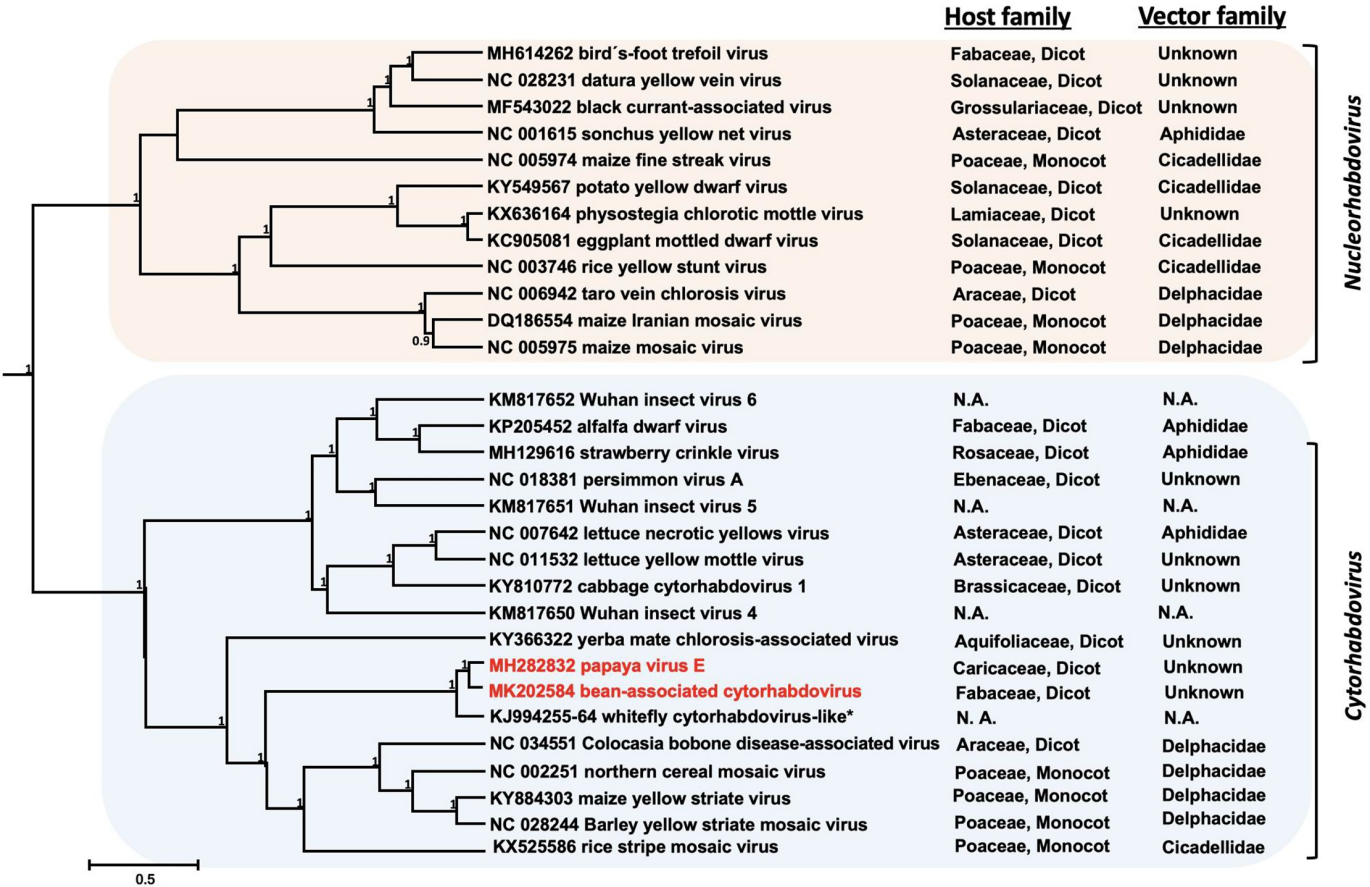
Multilocus phylogeny of monopartite plant infecting rhabdoviruses. Multiple sequence alignments of concatenated nucleocapsid, glycoprotein, and polymerase were analyzed in BEAST 1.8.4. Numbers above the nodes represent posterior probabilities. The two main clades corresponding to nucleorhabdoviruses and cytorhabdoviruses, respectively, are indicated by a colored rectangle. Genbank acc. numbers, host family and vector information (when available) are provided. N.A.: not applicable. Papaya virus E and bean-associated cytorhabdovirus are highlighted in red. *Based on rhabdovirus-like sequences annotated as part of whitefly genome.

The topology of the multi-locus tree was identical to the polymerase one and congruent with those inferred by analyses of the nucleocapsid and glycoprotein. Cyto- and nucleorhabdoviruses are monophyletic and each genus contains clades with well supported nodes corresponding with their vectors (Fig 2). For maize fine streak virus and rice yellow stunt virus, however, vector-associated phylogenies varied depending on the protein being analyzed (S2 Fig).

PpVE grouped with members of the *Cytorhabdovirus* genus, in a clade that includes the dicot-infecting YmCaV and CBDaV, and the monocot-infecting NCMV, MYSV, RSMV and barley yellow striate mosaic virus (BYSV), which are known (except for YmCaV) to be transmitted by Delphacidae/Cicadellidae vectors. Interestingly, virus-like sequences annotated as part of the *B*. *tabaci* genome grouped closely with PpVE and BaCV (Fig 2). This was supported by the high amino acid sequence identities observed between PpVE predicted proteins and orthologues from the *B*. *tabaci* genome (Table 2).

### Transmission electron microscopy (TEM)

**Fig 3:**
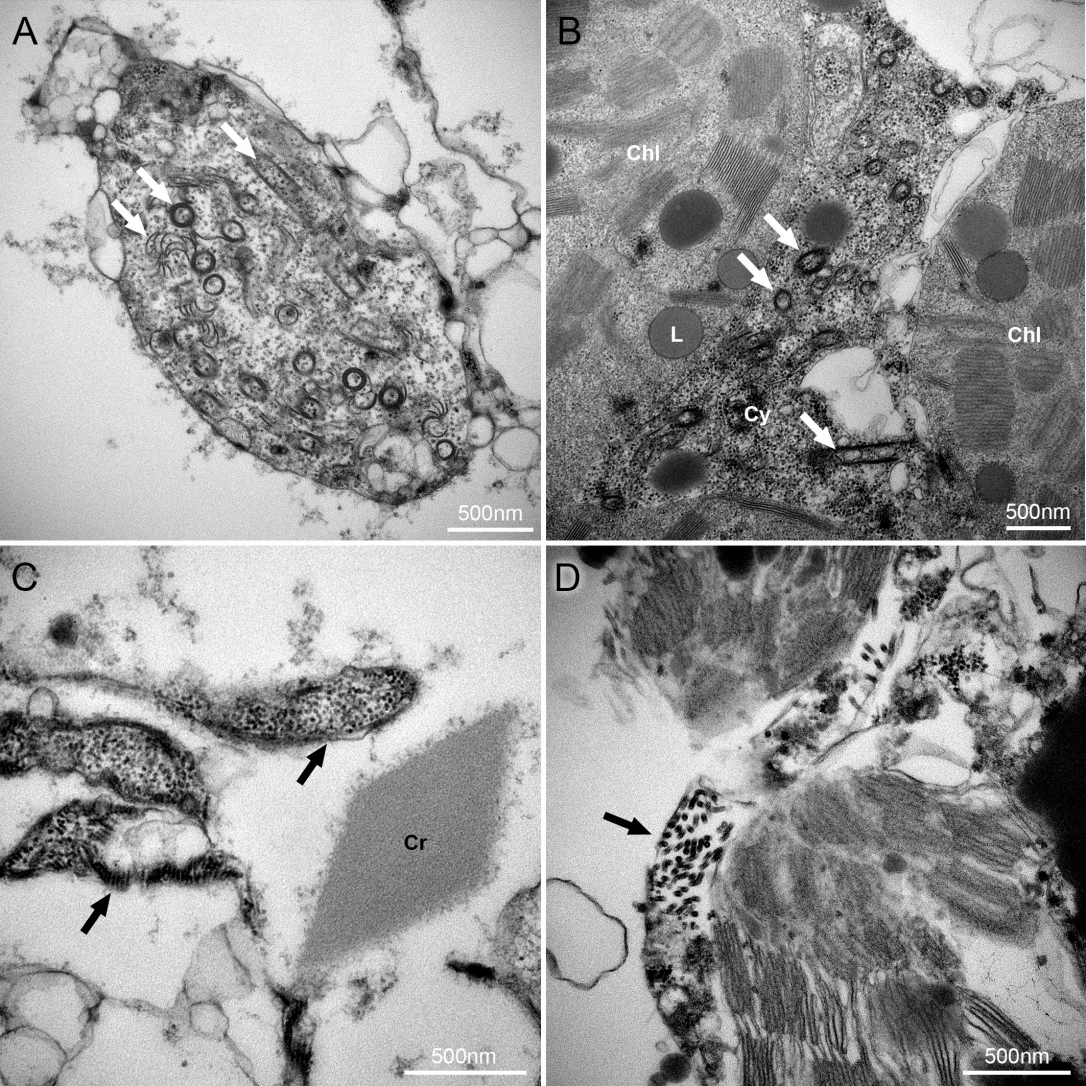
Transmission electron microscopy (TEM) images of the mesophyll cells of papaya leaves infected with papaya virus E and papaya ringspot virus. (A-B) White arrows showing potyvirus pin-wheel inclusions in three typical configurations in the cytoplasm. (C-D) Black arrows showing aggregations of rhabdovirus particles in the cytoplasm along with a crystalline inclusion. Chl: Chloroplasts; Cr: Crystalline inclusions; CW: Cell Wall; Cy: Cytoplasm; L: Lipid droplets.

TEM images of the mesophyll cells of papaya leaves infected with PpVE and PRSV are shown in Fig 3. Aggregations of rhabdovirus-like particles were detected in the periphery of chloroplasts. Pin-wheel and swirls, as well as crystalline inclusions typical of potyviruses, were readily observed. There were much fewer cells and aggregations of the rhabdovirus-like particles than of potyvirus, which could be related to virus titer. This observation is consistent with the HTS data, where 11.68% of the reads mapped to PRSV and only 0.23% to rhabdovirus.

### Virus survey

In Los Ríos, PpVE was found in 100% (n = 30) of samples (cv. Sunrise) collected from one-year old plants; whereas 13% (n = 30) were positive in an adjacent four-month-old field. In Guayas province, the virus was only detected in one out of 30 plants tested from a two-year-old field. In Manabí, 20% of plants tested positive from a three-year-old field (n = 30). The virus was not detected in selected fields of Sucumbíos, a forest province where ‘Criolla’ papaya was grown, or Santa Elena, where the Hawaiian cultivar Sunset was sampled.

All the plants that tested positive for the papaya cytorhabdovirus were also positive for PRSV. However, no differences in leaf symptoms were observed between PRSV-singly infected plants and plants co-infected with both viruses.

Genome diversity was inferred by comparing an 860 nt fragment of the virus polymerase. Five isolates from Los Ríos, five from Manabí, and only one from Guayas, were selected for RT-PCR. Sequence alignments of the amplified fragment showed a 99% identity across isolates from different provinces in Ecuador.

## Discussion

The *Rhabdoviridae* is one of the most diverse virus families as it contains viruses that infect arthropods, vertebrates and plants [10]. Here, we present the characterization of a new rhabdovirus discovered from papaya plants in Ecuador. Aligning entire genomes of plant rhabdoviruses is difficult due to high divergence of sequences. Nevertheless, the evolutionary history of the virus was confidently inferred using single or concatenated alignments of the nucleocapsid, glycoprotein and polymerase amino acid sequences.

The new virus, provisionally named papaya virus E (PpVE), grouped with members of the *Cytorhabdovirus* genus, with rice stripe mosaic virus, yerba mate chlorosis-associated virus and Colocasia bobone disease-associated virus as closest relatives.

In the concatenated alignment, 75.4% of the total length corresponded to the polymerase. The resulting tree was identical to the topology of the polymerase alone, supporting other studies that indicate using the polymerase is an accurate representation of the phylogeny for rhabdoviruses [38].

Furthermore, this study confirmed the strong correlation between phylogenetically-related species and their vectors [38]. Accordingly, PpVE is likely transmitted by a member of the *Delphacidae* or *Cicadellidae*. However, formal transmission experiments are needed to confirm this hypothesis. An interesting finding in this study was the genetic closeness (80% nucleotide identity) observed between PpVE and sequences from a whitefly genome annotated by Kumar and Upadhyay (unpublished, Genbank acc. numbers: KJ994255-KJ994264). We hypothesize that whiteflies used for the genomic analysis were either infected by a cythorhabdovirus or were carrying (potentially as a vector) a cytorhabdovirus acquired from a plant host.

The genome organization of rhabdoviruses includes five genes flanked by leader and trailer sequences at the 3’ and 5’ ends, respectively, resulting in the canonical arrangement: 3’-*l*-N-P-M-G-L-*t*-5’.

In plants, cyto- and nucleorhabdoviruses have an additional ORF between the P and M genes, whose product has cell-to-cell movement activity, resulting in the typical 3’-*l*-N-P-P3(MP)-M-G-L-*t*-5’ arrangement [13,14]. However, there are numerous examples of additional interspersed small ORFs of unknown functions [11,12,39].

In PpVE, two short ORFs (namely ORFs 4 and 5) were predicted with overlapping termination/initiation codons. This feature is commonly associated with a translation-reinitiation mechanism and has been documented, among others, for some animal rhabdoviruses [6]. The reinitiation mechanism is dependent on TURBS (*termination upstream ribosome binding site*), which includes a pentanucleotide motif that is complementary to the loop region of helix 26 of 18S rRNA [40]. TURBS-like motifs were not identified upstream the hypothetical reinitiation start codon of ORF 5 in PpVE. No homologues for PpVE ORFs 4 or 5 were found during this study. However, the two contiguous ORFs are flanked by conserved intergenic motifs (Fig 1), suggesting their expression.

One of the genomic hallmarks of rhabdoviruses is the presence of transcription regulatory signals, such as conserved intergenic motifs and self-complementary sequences located at terminal regions [10]. The genome of PpVE exhibits the conserved motif 3’-AUUCUUUUUG-5’ not only in intergenic regions (except in the P3(MP)-P4 junction), but also in the 5’ (*t*) region, supporting its involvement in transcription termination of the corresponding preceding gene.

In addition, a second conserved motif was detected in gene junctions P3(MP)-P4, P5-M and G-L of PpVE (Fig 1). Although the motif was not detected in gene junctions of closely related viruses, it was found partially conserved in a few junctions of some distantly related cytorhabdoviruses (Table 3). For instance, in maize yellow striate virus (MYSV), [41] such motif is present immediately after ORF 4, supporting the notion of its role in gene expression control.

This is the first report of a cytorhabdovirus in papaya and the first sequence deposited for any rhabdovirus of this host. Both its phylogenetic relatedness to cytorhabdoviruses and the TEM observations showing virus accumulation in the cytoplasm support the notion that this virus is not related to the papaya nucleorhabdoviruses reported in the early 1980s [16,17,18]. Since we could not find a papaya plant singly-infected with PpVE, symptomatology associated to the virus was not determined. Nevertheless, PpVE was detected in field plantings in three different provinces in Ecuador, strongly suggesting this is a naturally occurring virus. Comparisons among the polymerase sequence among 11 of these isolates were highly convergent (99% identity).

Lastly, in January 2019, the genome of BaCV was documented from Brazil [29]. BaCV shares 97% genome nucleotide sequence identity with and has similar genome organization to PpVE, except for lacking ORF 5 (Fig 1B). Given that the genome of PpVE has been available in Genbank since October 1, 2018 (acc. MH282832) and based on our data that indicate natural field spread, we propose that BaCV should be considered a bean-infecting strain of PpVE, classified in the newly proposed species Papaya cytorhabdovirus. This approach has already been supported by a letter to the editor [42].

## Supporting information

S1 Fig Amino acid sequence alignment of hypothetical P4 from bean-associated cytorhabdovirus (BaCV) and yerba mate chlorosis associated virus (YmCaV), with the concatenated P4 and P5 from the papaya virus E (PpVE). Yellow arrows denote P4 from BaCV or YmCaV; light-blue arrow indicates the concatenated P4-P5 in PpVE. Conserved residues are black-shaded. Percentage identities between PpVE and BaCV or YmCaV are indicated on the right.
(PNG)

S2 Fig Single protein phylogenies of the polymerase, glycoprotein and nucleocapsid of plant infecting monopartite rhabdoviruses. Arrow points viruses whose evolutionary history is not congruent among proteins. Numbers above the nodes represent posterior probabilities. Papaya virus E and bean associated cytorhabdovirus, which are 97% identical throughout the genome, are shown in red.
(PNG)
