# Supplementary figures and images for: Sequencing, genome analysis and prevalence of a cytorhabdovirus discovered in Carica papaya
Source: PLoS One. 2019 Jun 20;14(6):e0215798. doi: 10.1371/journal.pone.0215798 (PMC6586395; doi:10.1371/journal.pone.0215798)

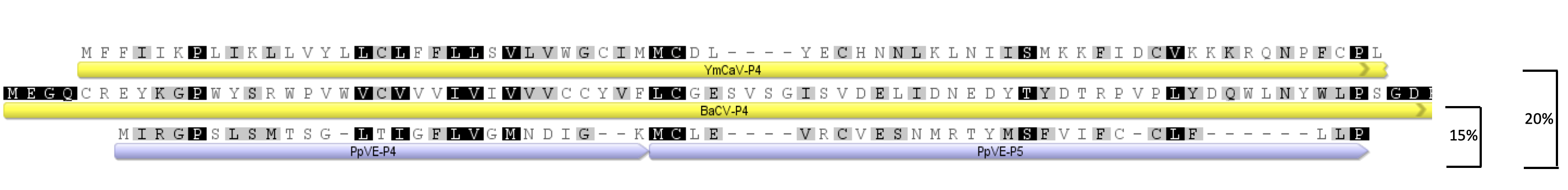

Supplement: S1 Fig — Yellow arrows denote P4 from BaCV or YmCaV; light-blue arrow indicates the concatenated P4-P5 in PpVE. Conserved residues are black-shaded. Percentage identities between PpVE and BaCV or YmCaV are indicated on the right. (PNG) [file pone.0215798.s001.png]

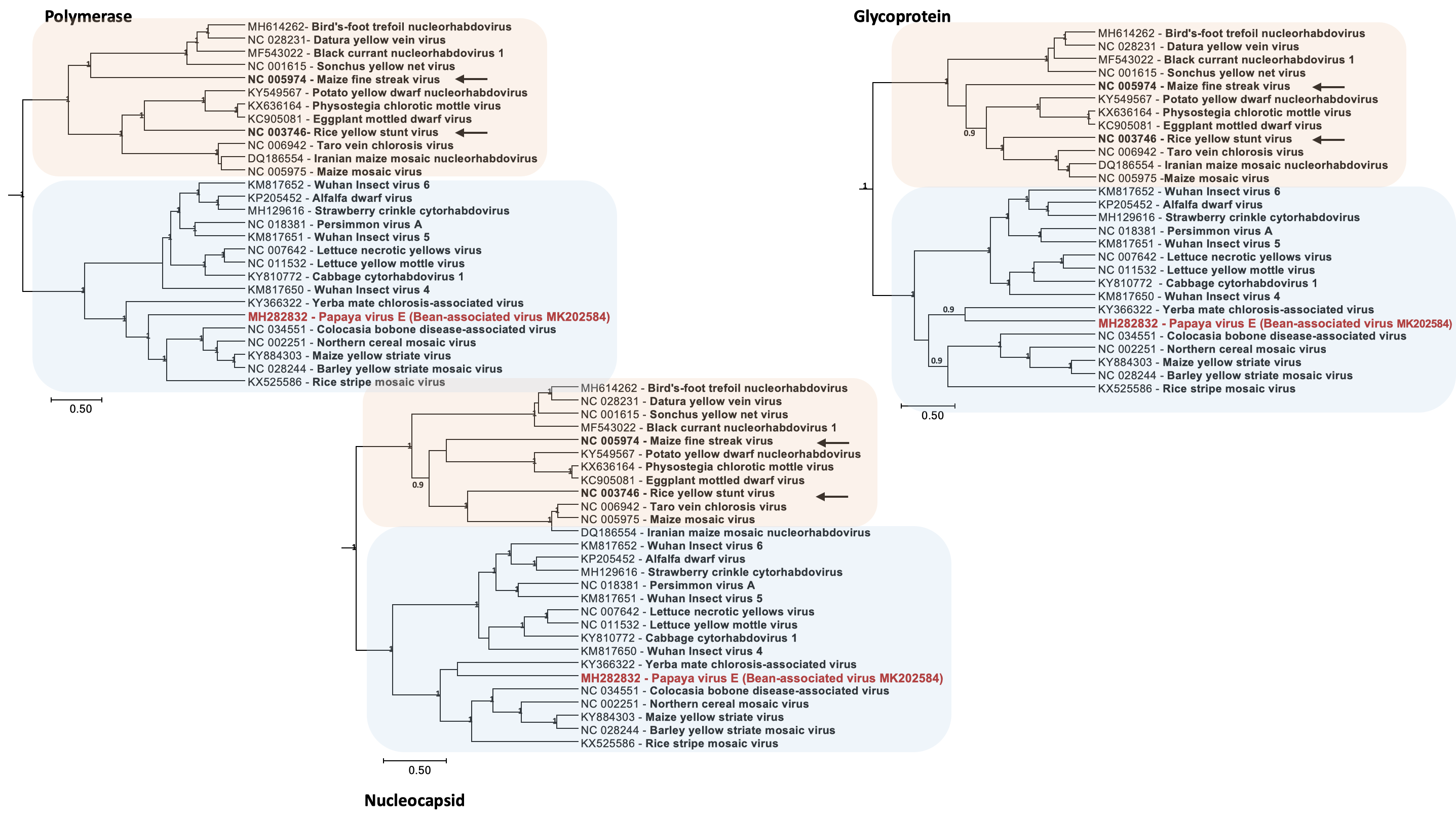

Supplement: S2 Fig — Arrow points viruses whose evolutionary history is not congruent among proteins. Numbers above the nodes represent posterior probabilities. Papaya virus E and bean associated cytorhabdovirus, which are 97% identical throughout the genome, are shown in red. (PNG) [file pone.0215798.s002.png]
